# Unilateral Partial Nephrectomy with Warm Ischemia Results in Acute Hypoxia Inducible Factor 1-Alpha (HIF-1α) and Toll-Like Receptor 4 (TLR4) Overexpression in a Porcine Model

**DOI:** 10.1371/journal.pone.0154708

**Published:** 2016-05-05

**Authors:** Zhiyong Zhang, Beatrice Haimovich, Young Suk Kwon, Tyler Lu, Billie Fyfe-Kirschner, Ephrem Odoy Olweny

**Affiliations:** 1 Division of Urology and General Surgery, Department of Surgery, Rutgers, Robert Wood Johnson Medical School, New Brunswick, NJ, United States of America; 2 Rutgers Cancer Institute of New Jersey, New Brunswick, NJ, United States of America; 3 Rutgers University, New Brunswick, NJ, United States of America; 4 Department of Pathology and Laboratory Medicine, Rutgers, Robert Wood Johnson Medical School, New Brunswick, NJ, United States of America; University of Pecs Medical School, HUNGARY

## Abstract

**Purpose:**

Ischemia/reperfusion (I/R) during partial nephrectomy (PN) contributes to acute kidney injury (AKI), which is inaccurately assessed using existent clinical markers of renal function. We evaluated I/R-related changes in expression in hypoxia inducible factor 1α (HIF-1α) and toll-like receptor 4 (TLR4), within kidney tissue and peripheral blood leukocytes (PBL) in a porcine model of PN.

**Materials and Methods:**

Three adult pigs each underwent unilateral renal hilar cross clamping for 180 min followed by a 15 min reperfusion. The contralateral kidney served as control. Biopsies of clamped kidneys were obtained at baseline (time 0), every 60 min during the hypoxic phase, and post-reperfusion. Control kidneys were biopsied once at 180 min. Peripheral blood was sampled at time 0, every 30 min during the hypoxic phase, and post-reperfusion. HIF-1α and TLR4 expression in kidney tissue and PBL were analyzed by Western blotting. I/R-related histological changes were assessed.

**Results:**

Expression of HIF-1α in clamped kidneys and PBL was below detection level at baseline, rising to detectable levels after 60 min of hypoxia, and continuing to rise throughout the hypoxic and reperfusion phases. Expression of TLR-4 in clamped kidneys followed a similar trend with initial detection after 30–60 min of hypoxia. Control kidneys exhibited no change in HIF-1α or TLR-4 expression. I/R-related histologic changes were minimal, primarily mild tubular dilatation.

**Conclusions:**

In a porcine model of PN, HIF-1α and TLR4 exhibited robust, I/R-related increases in expression in kidney tissue and PBL. Further studies investigating these molecules as potential markers of AKI are warranted.

## Introduction

Nephron-sparing surgery (NSS) is an important goal of kidney cancer surgery whenever feasible, as it better preserves renal function without compromising oncological efficacy in select clinical settings[1, 2]. However, NSS can cause acute kidney injury (AKI) resulting in the subsequent development of chronic kidney disease (CKD)[3, 4], albeit at lower rates than for nephrectomy. Mechanisms of NSS-induced AKI include loss of renal parenchymal volume, worsening of pre-existing CKD and ischemia-reperfusion injury (IRI)[5–7].

The literature on renal functional preservation following NSS is controversial. Retrospective clinical studies report minimal change in postoperative glomerular filtration rate (GFR) as assessed by serum creatinine (sCr)-based estimators[8]. Prospective clinical studies incorporating novel markers of AKI including neutrophil gelatinase-associated lipocalin (NGAL), report conflicting results with regard to estimation of AKI[9, 10]. Retrospective and prospective studies using ^99m^Tc-DTPA renal scintigraphy for split GFR estimation report a significant and lasting decrease in GFR of the operated kidney[6, 11, 12]. There is therefore a need for identification of sensitive and accurate molecular markers for the detection of early or subclinical AKI during NSS[13]. Such markers could facilitate earlier intervention to prevent or mitigate associated renal impairment, future research into the development of novel renoprotective agents, and development of surgical strategies aimed at minimizing renal impairment during NSS[13].

Previous studies in small animal models have suggested that hypoxia inducible factor-1α (HIF-1α) and toll-like receptor 4 (TLR4) mediate the kidney’s response to IRI[14, 15]. In this study, we investigated the feasibility of detecting acute changes in expression of HIF-1α and TLR4 in porcine peripheral circulation and kidney tissue during simulated partial nephrectomy (PN) with warm ischemia. We hypothesized that protein expression changes detected in the kidney might be reproduced in peripheral blood leukocytes (PBL), and if so, could serve as putative markers of AKI during kidney surgery.

## Materials and Methods

### Surgical procedures

The animal study protocol was approved by the Institutional Animal Care and Use Committee at Rutgers, Robert Wood Johnson Medical School. Three adult domestic female pigs, 50–70kg in size, were used. After an appropriate acclimation period, the animals were placed under general anesthesia using intramuscular injection of telazol (4.4 to 6.6 mg/kg), xylazine (1–2 mg/kg) and atropine (0.05 mg/kg), and maintained under inhalational anesthesia using isoflurane 2% in a 50/50 mixture of nitrous oxide and oxygen. Pulse oximetry levels were maintained at 98% or above. Intravenous access was obtained via an auricular vein and an arterial line was inserted in a carotid artery to facilitate precise blood pressure monitoring and serial blood draws. Intravenous hetastarch was administered at a rate of approximately 2L/hour to maintain hydration. A warming blanket was used to maintain body temperature at 36 to 38°C.

A baseline (time 0) blood sample of 6 ml was drawn into a heparin containing tube. Bilateral kidneys of each animal were then accessed retroperitoneally through separate flank incisions. One kidney served as the experimental kidney with the contralateral kidney serving as the control. Trans-peritoneal access was specifically avoided so as to minimize potential for data confounding by unrecognized ischemia of intra-abdominal organs during surgical manipulation. After surgical exposure of each kidney, a thin wedge biopsy of the experimental kidney was obtained sharply (time 0). Hemostasis was obtained using oxidized cellulose bolsters and 0-vicryl renorrhaphy sutures for parenchymal repair. The renal hilum of the experimental kidney was then cross-clamped for a total of 180 minutes followed by a 15-minute reperfusion. Additional blood samples (6 ml each) were obtained from the arterial line at 30, 60, 90, 120, and 180 minutes during the hypoxic phase, and following reperfusion (195 minutes). Serial wedge biopsies of the experimental kidney were obtained during the hypoxic phase at 60, 120 and 180 minutes, and post-reperfusion (195 minutes). Hemostasis was obtained after each biopsy as described above. A single wedge biopsy was obtained from the control kidney at 180 minutes, after which both kidneys were excised and fixed in formalin.

All blood samples were processed immediately for leukocyte isolation as described by Zhang *et al*.[16] Kidney biopsies were flash-frozen in liquid nitrogen and subsequently stored at -80°C pending processing for Western blotting. Animals were euthanized by first inducing deep anesthesia using intravenous pentobarbitol (100 mg/kg), followed by an intravenous bolus of saturated potassium chloride (30 ml).

### Samples processing for Western blot analysis

Blood samples were processed as described: the blood was first mixed with lysis buffer (bicarbonate-buffered ammonium chloride solution) at a ratio of 20:1. Once the erythrocytes lysed, the samples were centrifuged for 10 minutes at 400 g. The leukocyte pellet was washed once with phosphate buffered saline. After centrifugation, the leukocyte pellet was lysed in RIPA buffer (1% Triton X-100, 1% deoxycholic acid, 10mM Tris-HCl, pH 7.2, 158 mM NaCl, 0.1% SDS, and 1 mM PMSF) and Roche complete protease inhibitor cocktail^12^. The lysates were stored at -80°C. Sections of kidney biopsy samples were similarly lysed in RIPA buffer. PBL and kidney tissue RIPA-lysates were normalized for protein content, and then were subjected to western blot analysis, probing with antibodies to HIF-1α (Santa Cruz, SC-10790), TLR4 (Santa Cruz SC-10741) and actin (Sigma, A2066). Western blots were run in duplicate.

### Histological evaluation

Portions of the kidney wedge biopsies obtained at times 0, 60, 120, 180 and 195 minutes were separately submitted for H&E staining, and were evaluated by an experienced pathologist blinded to the tissue source.

## Results

**Fig 1:**
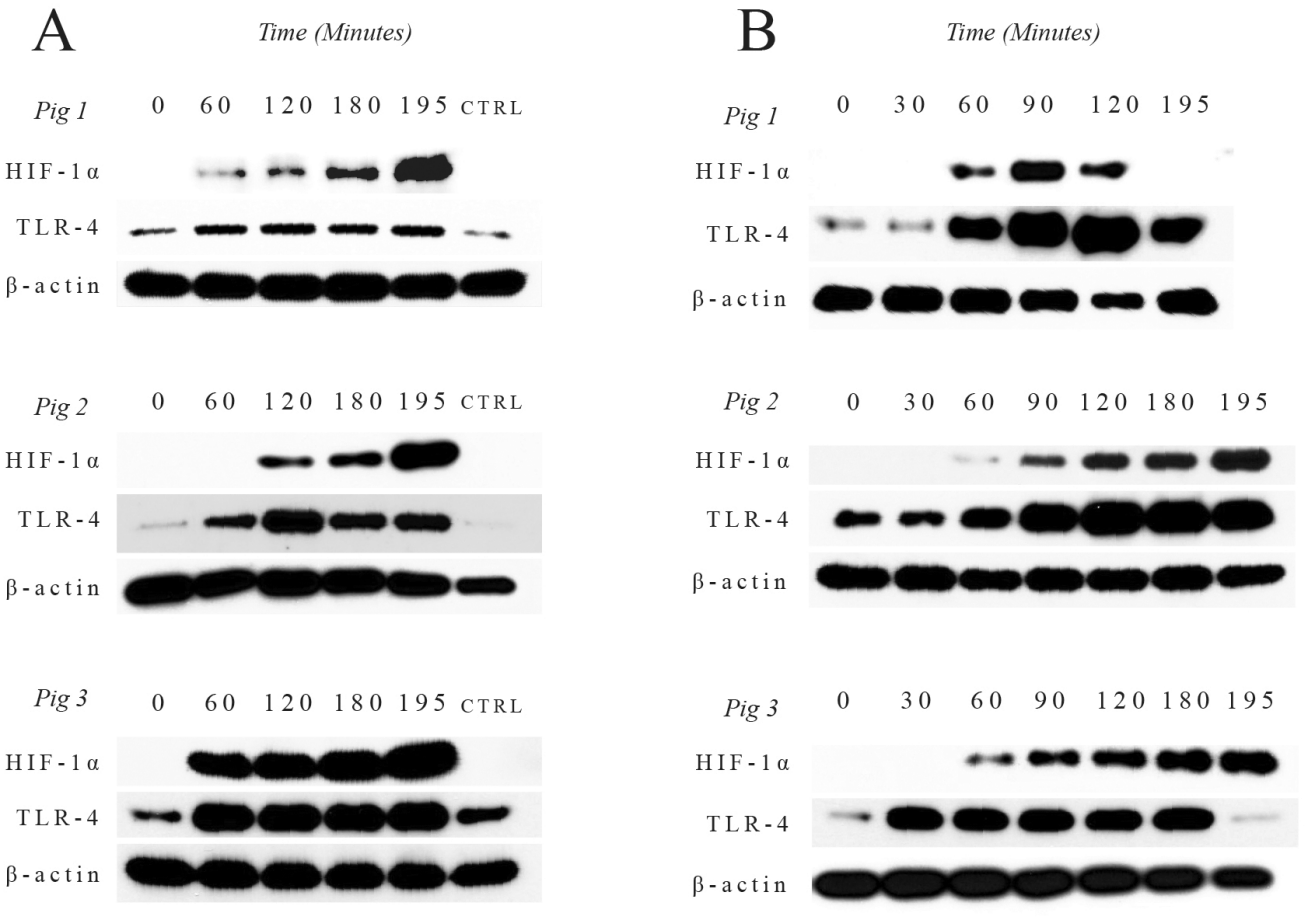
Western blots of hypoxia inducible factor-1α (HIF-1α) and toll like receptor 4 (TLR4) expression in kidney tissue (A) and peripheral blood leukocytes (B). CTRL = control. Beta-actin (β-actin) was used as a loading control.

All surgeries were completed without complications. In kidney tissue lysates, the expression of HIF-1α was below detection levels at time 0. During the hypoxic phase, HIF-1α expression in cross-clamped kidneys was detectable by 60 min, and its level continued to rise throughout the ischemia interval and post reperfusion. HIF-1α expression remained below detection levels in control, unclamped kidneys. A similar HIF-1α expression pattern was observed in PBL; HIF-1α expression in PBL was below detection level at baseline (time 0), became detectable by 60 minutes after unilateral cross-clamping, and continued to rise throughout the remainder of the study (Fig 1).

The changes in TLR4 expression exhibited similar trends to those of HIF-1α in both kidney tissue lysates and PBL. TLR4 expression in bilateral kidneys and PBL was low at baseline (time 0). During the hypoxic phase, there was a notable increase in TLR4 expression in cross-clamped kidneys as well as in PBL after 30–60 minutes of ischemia, with continued rise throughout the remainder of the study. In marked contrast, TLR4 expression in the control un-clamped kidney did not change over time (Fig 1). Collection of a blood sample from Pig 1 at 180 minutes was inadvertently overlooked, and therefore values for expression of HIF-1α and TLR4 expression in PBL are missing for this time point.

**Fig 2:**
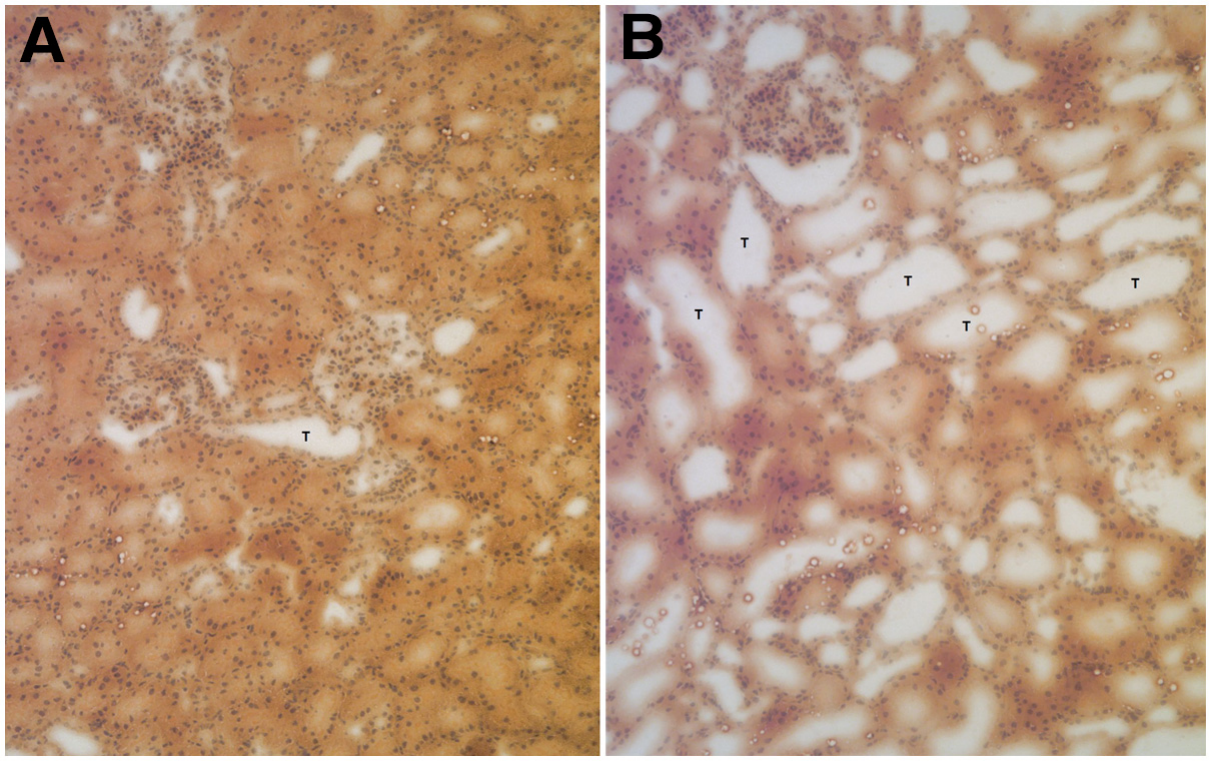
Representative hematoxylin and eosin (H&E)-stained biopsy sections from controls (A) and experimental (cross-clamped) kidneys (B) at 10x magnification. T = tubules. Histologic change in hypoxic kidneys primarily consisted of tubular dilation without associated inflammation.

On blinded histologic examination of H&E-stained kidney biopsy specimens, there was minimal histologic change from baseline (time 0) in the biopsy specimens obtained at 60, 120 and 180 minutes during the hypoxic phase, or post-reperfusion (195 min). In addition, there was minimal histologic difference between biopsies from the cross-clamped and the unclamped control kidneys. The main histologic change in cross-clamped kidneys was mild tubular dilatation consistent with acute tubular injury. There was no evidence of inflammation in the biopsy specimens from either cross-clamped or unclamped control kidneys (Fig 2).

## Discussion

The impact of kidney surgery on the development new onset CKD, or the worsening of pre-existent CKD, is well documented. Kidney removal results in the greatest deterioration in post-operative kidney function[17]. However, up to 20% of patients undergoing nephron-sparing surgery also exhibit measurable decline in kidney function, depending on the amount and/or quality of preserved renal parenchyma and warm ischemia time[18]. Advanced stage CKD has been linked to increased risk of mortality, cardiovascular events and hospitalization[19], making functional preservation an important goal of kidney cancer surgery.

AKI is a clinical syndrome characterized by acute deterioration in kidney function resulting in a wide spectrum of clinical manifestations[20]. Permanent structural and functional damage occurs in 50% of patients with AKI, including subsequent development of CKD or ESRD[21]. Warm ischemia and direct injury to functioning renal tissue are significant contributors to AKI during nephron-sparing renal surgery. Current sCr-based clinical estimators of kidney dysfunction do not reliably estimate the degree of AKI for several reasons. First, they lag the acute changes at the cellular level by several days, and may not detect injury until a steady state has been reached, typically several days after the injury. Second, since creatinine is both filtered and secreted by the kidney, sCr-based estimators of kidney function tend to overestimate renal function, thus masking the true severity of AKI. Third, in the setting of a normal contralateral kidney, sCr-based estimators further underestimate AKI due to compensatory changes by the normal kidney[18, 22]. Accordingly, numerous alternative potential biomarkers of kidney injury have been evaluated over the past 2 decades, virtually all of them being urine based[13]. Clinical implementation of these novel biomarkers is limited to date, largely due to limited availability of the markers, as well as poor sensitivity and specificity in urologic patients[23].

In this pilot study, we investigated the feasibility of measuring acute HIF-1α and TLR4 expression in the peripheral circulation of pigs subjected to unilateral, ischemia-induced renal injury. HIF-1α and HIF-1β form the transcription factor HIF-1 complex. Whereas HIF-1β is expressed constitutively, the expression of HIF-1α is highly regulated and commonly stabilized under hypoxic conditions. However, stressors that activate TLR4 stabilize the expression of HIF-1α under either hypoxic or normoxic conditions[24–26]. Studies have suggested that HIF-1α/HIF-1, which can be detected in transplanted kidneys, protects against IRI[27]. Experiments in post-transplant human biopsies and in murine models of IRI have similarly suggested that HIF-1α accumulates during post-ischemia reperfusion[14].

Toll-like receptors (TLRs) play a key role in innate immunity and tissue inflammation. TLRs are activated by pathogen-associated molecular patterns (PAMPs) as well as endogenously released danger-associated molecular patterns (DAMP’s)[28, 29]. Studies in mice showed that TLR4 activation occurs in response to renal damage during hypoxia. More specifically, downstream signaling results in heightened inflammation, amplifying the initial IRI-mediated renal damage[15, 30].

Our data demonstrate that HIF-1α and TLR-4 are acutely overexpressed in hypoxic kidneys and PBL during warm ischemia and post-reperfusion. To our knowledge, this is the first study to demonstrate *in vivo* expression of these molecules in a large animal model during active tissue hypoxia. These findings are significant since the porcine kidney is similar to the human kidney in genetics, development, size, anatomy and physiology[31]. Based on this, we speculate that the responses observed in the porcine model of PN also unfold in human kidney under hypoxia. However, a notable difference between the pig and human kidney is that the pig kidney is significantly more resilient to ischemic stress. In solitary kidney models, pig kidneys were found to recover from ischemic insults of up to 90 minutes, with a maximal tolerance limit of 120 minutes[32, 33]. These tolerance limits were determined by evaluating renal function at several postoperative intervals out to 15 days. In the present proof-of-concept study, the ischemic window was extended to 180 min to ensure occurrence of clinically significant ischemic renal injury. We observed only minimal histologic change by H&E staining after 180 minutes of ischemia followed by a brief re-oxygenation. In marked contrast, within PBL, increased expression of HIF-1α or TLR-4 was observed after 60 minutes or 30–60 minutes of ischemia respectively, well within the tolerable ischemic limit for pig kidneys. The changes in HIF-1α and TLR4 expression observed in PBL paralleled the changes observed in the kidneys subjected to hypoxia. Since similar changes were not detected in the unclamped control kidney, these data suggest the changes in HIF-1 α and TLR4 expression detected in PBL were caused by renal hypoxia.

A possible explanation for why molecular changes within hypoxic kidney tissue were detectable in circulating blood may be the existence of pre-existing collateral circulation between the kidney parenchyma and extra-renal blood vessels. Indeed, studies in dogs and humans have demonstrated evidence of such collateral circulation, which plays a role in renal parenchymal preservation after renal artery occlusion[34, 35]. Although we did not check for the presence of collateral circulation in our model, we postulate that similar to other species, collateral renal circulation exists in pigs. Differences in collateral circulation patterns could also explain the minor variations between the animals in HIF-1 α and TLR4 expression levels at specific time points. Hessel *et al*. reported that collateral circulation varied between different vascular beds even within the same species[35]. However, the overall expression pattern in HIF-1 α and TLR4 in response to renal ischemia was similar for each animal.

Conde *et al*. described the role of increased HIF-1α expression during renal ischemia/reperfusion stress in a study conducted in three different settings: human proximal epithelial cells, Sprague-Dawley rat kidneys, and post-transplant human renal allograft biopsies[14]. They determined that HIF-1α in proximal renal tubular cells is expressed in a biphasic fashion, during hypoxia and during re-oxygenation, both *in vitro* and *in vivo*. HIF-1α was found to mediate proximal renal tubular cell survival and recovery following IRI. Indeed, analyses of the renal allograft biopsies revealed a significant negative correlation between HIF-1α expression and ATN severity[14].

In contrast to the renoprotective role of HIF-1α, increased TLR4 expression in response to ischemia further amplifies the initial damage via activation of inflammatory mediators[15, 30]. Using a mouse model, Wu *et al*. found that TLR4 expression is significantly increased on days 1 and 3 after ischemia/reperfusion[15]. An interesting finding in our study was that TLR4 expression is upregulated as early as 30 minutes after renal hypoxia in the pig kidney. We therefore infer that TLR4-mediated immune system activation occurs early during tissue hypoxia; this could be a potential target for future intervention to minimize IRI-mediated AKI during renal surgery, pending further investigation.

Studies have suggested a possible link between HIF-1α and TLR4. In an *in vitro* study, Kim *et al*. found that hypoxic stress up-regulates the expression of TLR-4 in macrophages via a HIF-1α-dependent mechanism[36]. Zhou *et al*. demonstrated that downstream TLR4 signaling leads to the accumulation of HIF-1α *in vivo*, during lung ischemia-reperfusion injury[37]. Although the precise interactions between these molecules are beyond the scope of our study, it is likely that further complex mechanisms beyond those described above could be at play during the kidney response to ischemia/reperfusion. This warrants further investigation.

A few limitations of our study warrant discussion. First, the number of animals used was small. However, this was a feasibility study, and we believe that the small sample size was justified for establishing proof-of-concept at minimal cost and at minimal adverse impact to animal welfare. Secondly, this was a non-survival experiment, thereby limiting assessment of post-operative renal function. Thus, it is presently unclear how increased expression of HIF-1α and TRL4 during renal ischemia/reperfusion correlates with postoperative renal function. Further studies are underway to address these issues. Third, the experiments were conducted in normal, healthy kidneys and it is uncertain whether similar findings would be observed in cancer-bearing kidneys.

## Conclusion

In this feasibility study, we demonstrate for the first time that increased expression of HIF-1α and TLR4 occurs during renal ischemia/reperfusion in a large animal model of partial nephrectomy, and that this increased expression is detectable in both ischemic renal tissue and in peripheral blood leukocytes. Pending further studies investigating the correlation of these findings with postoperative renal functional outcomes, these molecules have the potential for future development as biomarkers of AKI during renal surgery and/or to serve as targets for intervention to minimize the deleterious effects of IRI on postoperative renal function.
